# Preparation and Characterization of Porous Materials from Pineapple Peel at Elevated Pyrolysis Temperatures

**DOI:** 10.3390/ma15134686

**Published:** 2022-07-04

**Authors:** Wen-Tien Tsai, Raquel Ayestas, Chi-Hung Tsai, Yu-Quan Lin

**Affiliations:** 1 Graduate Institute of Bioresources, National Pingtung University of Science and Technology, Pingtung City 912, Taiwan; wsx55222525@gmail.com; 2 Department of Tropical Agriculture and International Cooperation, National Pingtung University of Science and Technology, Pingtung City 912, Taiwan; raquelayestas@yahoo.com; 3 Department of Resources Engineering, National Cheng Kung University, Tainan 701, Taiwan; ap29fp@gmail.com

**Keywords:** pineapple peel, pyrolysis, biochar, pore property, surface chemistry, adsorption test

## Abstract

In this work, pineapple peel (PP) was reused as a precursor in biochar (BC) production at elevated temperatures (i.e., 500–900 °C) for residence times of 0–60 min. The findings showed that pyrolysis temperature and residence time played a vital role in pore development. As pyrolysis temperature increased from 800 to 900 °C for residence times of 20 and 60 min, the data on the Brunauer–Emmett–Teller (BET) surface area of the resulting biochar products significantly jumped from 11.98–32.34 to 119.43–133.40 m^2^/g. In addition, there was a significant increase in the BET surface area from 1.02 to 133.40 m^2^/g with the residence time of 0 to 20 min at 900 °C. From the data of the nitrogen adsorption–desorption isotherms and the pore size distribution, both micropores (pore diameters of <2.0 nm) and mesopores (pore diameters of 2.0–50.0 nm) are present in the PP-based biochar products. Due to its good fittings in the pseudo-second-order model and its hydrophilic nature, as seen in the Fourier transform infrared spectroscopy (FTIR), the resulting biochar could be a porous material to be used for the effective removal of cationic compounds (i.e., methylene blue (MB)) from liquid phases.

## 1. Introduction

Pineapple (*Ananas comosus*) is an herbaceous, tropical, and monocot perennial plant, and is an edible fruit [1]. In order to meet market demand and consumer convenience, pineapple fruits are generally consumed fresh by removing the peel and cutting the fruit (or pulp) to pieces and processing it into canned/packaged products. This popular fruit is cultivated extensively in the tropical and subtropical countries globally. In 2020, the world production of pineapples amounted to around 28 million metric tons [2]. Generally, pineapple is harvested in fields by removing its non-edible parts. such as its leaves and crown. The harvested pineapple must be further peeled before eating or canning. Therefore, a massive amount of pineapple peel is generated in kitchens, markets, and food-processing factories [3]. Due to its high contents in moisture, biological organics, and lignocellulose, the biomass waste could cause environmental problems during clearance (storage and transportation) and treatment (disposal and incineration). For example, treatment by sanitary landfill not only results in the emission of greenhouse gases (GHGs) and odorants, it also facilitates large amounts of leachate which could lead to surface water pollution. To minimize the environmental impact and promote the circular economy, the valorization of pineapple peel has been exploited and reviewed by researchers in recent years [3,4,5,6].

The pineapple fruit is famous for its high richness in functional ingredients such as polyphenols, pectin, cellulose, and sugars (carbohydrates). In this regard, pineapple peel, accounting for about one-third of a pineapple’s total weight [7], is often processed to produce bioactive compounds and organic products, including phenolic antioxidants, organic acids, ethanol (wine), dietary fiber, proteolytic enzymes (bromelain) [3,4,5,6], special sugars [8], and lignocellulose nanocrystals [9]. On the other hand, the dried biomass waste also contains a lot of lignocellulosic constituents, which are related to contain oxygen-containing functional groups for providing complexation and ion exchange [10]. Therefore, pineapple peel has the potential to be reused as a biosorbent in the removal of cationic pollutants, including zinc [11,12,13], cadmium [14], nickel [15], and dye [16], from aqueous solutions. However, its maximal uptake capacities have been highly associated with its physical properties such as surface porosity and particle size. Ahmad et al. [17] further investigated chemically oxidized pineapple fruit peel biomass for the removal of cadmium and lead ions from aqueous solutions, and it showed enhanced removal efficiencies likely due to the introduction of carboxylic and hydroxyl groups onto the surface of the modified biomass.

It is well known that biochar is a carbon-rich material formed at >300–400 °C under limited or oxygen-deficient conditions [18]. In order to enhance its pore properties and/or adsorption performance, there are limited studies that have focused on the preparation of biochar from pineapple peel (PP) [19,20,21,22,23,24]. Fu et al. [19] prepared PP-based biochar products at three temperatures (350, 500, and 650 °C) with a heating rate of 10 °C/min for 3 h, and they showed an increasing trend for its pore properties based on the Brunauer–Emmett–Teller (BET) surface area (i.e., it increased from 0.82 to 6.64 m^2^/g). In the study by Wang et al. [20], PP was heated at different temperatures (350, 500, and 750 °C) in a muffle oven for 2 h with a heating rate of 5 °C/min. The data showed that the BET surface area of the tested biochar also increased from 0.76 m^2^/g (350 °C) and 2.16 m^2^/g (500 °C) to 328.80 m^2^/g (750 °C). According to the experiments by Shakya and Agarwal [21], a PP sample was heated from room temperature to the desired temperatures (350, 450, 550, and 650 °C) with a heating rate of 5 °C/min and a holding time of 60 min. The BET surface area values indicated a fluctuated variation, ranging from 3.37 m^2^/g to 11.11 m^2^/g. Hu et al. [22] investigated the biochar products prepared from three species of fruit peel (orange, pineapple, and pitaya) at 300, 400, 500, and 600 °C with the residence times of 2 h and 4 h, which were used to evaluate the characteristics and sorption for ammonium. However, the resulting biochar products had small BET surface area values, which ranged from 1.27 m^2^/g to 1.69 m^2^/g. Mahmuda et al. [23] produced the activated carbon and biochar from pineapple waste biomass for the removal of methylene blue. The latter was obtained under the pyrolysis conditions of 340 °C for 2 h and had a BET surface area value of 56.38 m^2^/g. Otieno et al. obtained biochar with a BET surface area of 140 m^2^/g using ≤300 μm under vacuum carbonization at 400 °C for 4 h [24], and it was used for removing ammonium nitrogen from human urine solutions.

Among the process parameters in the pyrolysis system, treatment temperature and residence time have the greatest influence on the pore properties of the resulting biochar [25,26]. Obviously, the pyrolysis of PP for producing biochar has been adopted at lower temperatures (˂750 °C) and longer residence times (>1 h) in the literature. Therefore, the aims of the present study were to produce porous PP-based biochar products at elevated pyrolysis temperatures (i.e., 500–900 °C) under lower residence times (i.e., 0–60 min). The pore and chemical characteristics of the resulting biochar products were characterized by using the nitrogen adsorption–desorption method, scanning electron microscope—energy dispersive X-ray spectroscopy (SEM-EDS), and Fourier infrared spectrometer (FTIR). Furthermore, the adsorption performances of the optimal biochar for removal of methylene blue (MB) from water were tested under various initial MB concentrations.

## 2. Materials and Methods

### 2.1. Materials

The biomass PP was collected from a local pineapple farm nearby Neipu Township (Pingtung City, Taiwan). This PP-derived feedstock was dried in two steps: it was dewatered under the sun, and then it was dried in the oven at 105 °C. Once the peels were completely dry, they were pulverized and sieved. Medium-sized particles with the range of mesh numbers 20 (sieve opening = 0.168 mm) and 12 (sieve opening = 0.841 mm) were collected and stored in the oven at 60 °C to avoid moisture adsorption. The dried PP was subsequently used to perform the thermochemical analyses and the pyrolysis experiments. On the other hand, methylene blue (MB) was probed as a target adsorbate in the aqueous solution for determining the adsorption performance of PP-based biochar. It was purchased from Merck Co. (Darmstadt, Germany).

### 2.2. Thermochemical Properties of Pineapple Peel

According to previous reports [27,28] and other studies [29,30,31], thermogravimetric analysis (TGA) is a powerful tool for determining the pyrolysis characteristics of lignocellulosic biomass. Herein, the thermochemical properties of the dried PP, including TGA, proximate analysis (i.e., ash, volatile matter, and fixed carbon), energy dispersive X-ray spectroscopy (EDS), and calorific value, were first performed. The authors used the following analytical instruments:

- TGA: model TGA-51 (Shimadzu Co., Tokyo, Japan)
- EDS: model X-stream-2 (Oxford Instruments plc, Abingdon, UK)
- Calorific value: model CALORIMETER ASSY 6200 (Parr Co., Moline, IL, USA)

Herein, the TGA was performed from 25 °C to 900 °C at a heating rate of 10 °C/min under a nitrogen flow. Based on the original thermogravimetric data, the derivative thermogravimetry (DTG) was plotted against the temperature for obtaining useful information about the thermal decomposition mechanism of the lignocellulosic sample.

### 2.3. Pyrolysis Experiments

Based on the data from the TGA, the preparation of biochar from PP (approximately 3 g for each experiment) was set at higher pyrolysis temperatures (i.e., 500, 600, 700, 800, and 900 °C) using a heating rate of about 10 °C/min in a vertical fixed-bed furnace for various residence times (i.e., 0, 20, 40, and 60 min) under a nitrogen atmosphere (500 cm^3^/min) [27,32]. The biochar products were denoted as PP-BC-temperature-time. For example, PP-BC-900-20 correspondingly indicated the preparation of pineapple peel biochar at the pyrolysis temperature of 900 °C for 20 min. In addition, the biochar yield was given by the ratio of the dry mass of the resulting biochar from the furnace to the dry mass of PP loaded into the furnace. Each pyrolysis experiment was performed in duplicate.

### 2.4. Analysis of Resulting Biochar Properties

The pore properties of the resulting biochar were determined by the N_2_ adsorption–desorption isotherms at −196 °C using an accelerated surface area and a porosimetry system (model: ASAP 2020; Micromeritics Co., Norcross, GA, USA) [33]. Before starting the measurement, the biochar sample (0.25–0.30 g) was degassed at 250 °C for 3 h in a vacuum operation. Herein, the specific surface area was obtained from the commonly used models, including the single-point, Brunauer–Emmett–Teller (BET), and Langmuir. The *t*-plot method was used to determine the micropore surface area and micropore volume [34]. The surface functional groups of the resulting biochar product were analyzed by the Fourier transform infrared (FTIR) system (model: FT/IR-4600; JASCO Co., Tokyo, Japan), which were scanned in the range of 400–4000 cm^−^^1^ by a 16 cm^−^^1^ resolution. In order to obtain the peaks of the function groups more sharply, the ground biochar sample was pre-mixed with IR-grade potassium bromide (KBr) to form a fine powder of approximately 0.1 wt%, which was then pressed into a pellet disc by a hydraulic press. However, the elemental compositions of the resulting biochar products were determined by EDS, which has been described in Section 2.2. Further, the porous texture of the resulting biochar product was detected by a tabletop scanning electron microscope (SEM) (model: TM3030; Hitachi Co., Tokyo, Japan).

### 2.5. Adsorption Experiments

In order to test the adsorption performance of the optimal biochar product (i.e., PP-BC-900-20), the batch adsorption experiments were performed in a 3 L agitation tank [35], which was immersed in a refrigerated circulator. In the present study, the adsorption uptakes (or removals) of MB by the biochar adsorbent from the aqueous solution (2 L) were obtained at the initial MB concentrations of 5 and 15 mg/L under fixed conditions (i.e., a solution temperature of 25 °C, biochar dosage of 0.3 g/2 L, and agitation speed of 200 rpm). During the experiments, an aliquot (about 10 cm^3^) was drawn from the solution at specified intervals (i.e., 1, 5, 10, 20, 30, 40, 50, and 60 min). The samples were then filtered through a mixed cellulose esters membrane filter with size of 25 mm before analysis. The spectroscopy method was used for MB detection on a UV–Vis spectrophotometer (model: UH5300; Hitachi Co., Tokyo, Japan) at the maximum absorption wavelength (i.e., 661 nm). The removal efficiency (R, %) and adsorption capacity (*q_t_*, mg/g) of the biochar for MB at time *t* were calculated as:

R = [1 − (C*_t_*/C_0_)] × 100

*q_t_* = (C_0_ − C*_t_*)/m × V

where C_0_ (mg/L) and C*_t_* (mg/L) represent the concentrations of MB at the initial and time *t* (min), respectively, while m (g) is the mass of biochar adsorbent (0.3 g) and V (L) is the volume of MB-containing solution (2 L). Furthermore, the pseudo-second-order model was adopted to quantitatively compare the apparent adsorption kinetics by using its linear form [36]:

*t*/*q_t_* = 1/(*k* × *q_e_*^2^) + (1/*q_e_*) × *t*

where *q_t_* is the adsorbed MB amount at interval time *t* (mg/g), *q_e_* is the adsorbed MB amount at equilibrium (mg/g), and *k* is the adsorption rate constant of this model (g/mg·min). Using the fitted parameters of this model, the time of half of the adsorption at equilibrium (*t*_1/2_) and the initial adsorption rate (*h*) can be calculated by the following equations [37]:

*t*_1/2_ = 1/(*k* × *q_e_*)

*h* = *k* × *q_e_*^2^

## 3. Results and Discussion

### 3.1. Thermochemical Properties of Pineapple Peel

The proximate analysis, elemental composition, and calorific value of the dried PP are summarized in Table 1. Obviously, the PP biomass has a relatively higher ash content (8.28 wt%) than those of common biomass residues [38], but it was close to other studies [23,39]. Typically, the ash values of biomass husks (or shells) are in the range of 1.4–8.3 wt% on a dry basis [40], although the higher ash contents may be found in rice husk and cocoa pad husk [41]. Ash content represents the incombustible remaining after a biomass sample is completely burned. Therefore, the calorific value of the dried PP (18.02 MJ/kg) was a slightly lower than those of other biomass husks [38]. By comparison, the ash content and calorific value of the dried rice husk were 16.46 wt% and 13.96 MJ/kg, respectively [42]. In addition, this energy content was consistent with its high content of carbon (about 50.5 wt%), volatile matter (73.23 wt%), and fixed carbon (18.49 wt%), as listed in Table 1.

**Figure 1:**
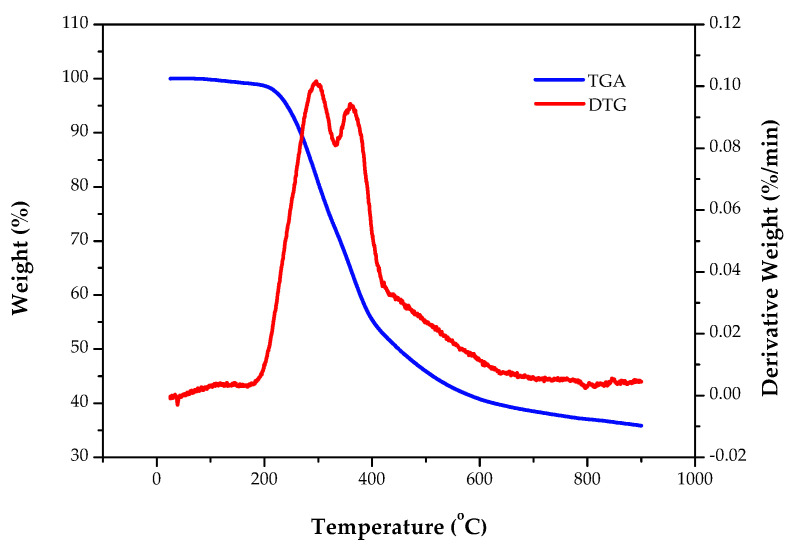
TGA and DTG of pineapple peel (PP) at temperature increases of 10 °C/min under a N_2_ gas flow.

Figure 1 depicts the thermogravimetric analysis (TGA) and derivative thermogravimetry (DTG) curves of the dried PP at 10 °C/min under an N_2_ flowrate of 50 cm^3^/min. Obviously, the initial weight decline occurred in the range of 25–200 °C due to the removals of bound water and low-molecular-weight volatiles. The significant weight loss was observed in the range of 200–400 °C, which further showed two peaks of degradation at 350 °C and 385 °C on the DTG curve. The former between 200 °C and 330 °C should be due to the thermal degradation of hemicelluloses because it is of a lower thermal stability than cellulose and lignin [18]. The latter between 330 °C and 420 °C could be mainly caused by the cellulose. When the pyrolysis temperature was heated above 450 °C, the gradual weight loss could be attributed to the carbonization (or charring) of complex lignocellulosic fractions (e.g., lignin) and the volatilization of low-melting-point minerals (or inorganics). Based on the data in Figure 1, the pyrolysis experiments were performed from 500 to 900 °C at a heating rate of 10 °C/min for producing biochar with higher pore properties such as specific surface area and pore volume [43]. In general, the amorphous carbon matrix in charred biomass will be gradually lost and porosity will begin to develop at higher pyrolysis temperatures (>500 °C) [44].

### 3.2. Yield and Pore Analysis of the Resulting Biochar Materials

**Figure 2:**
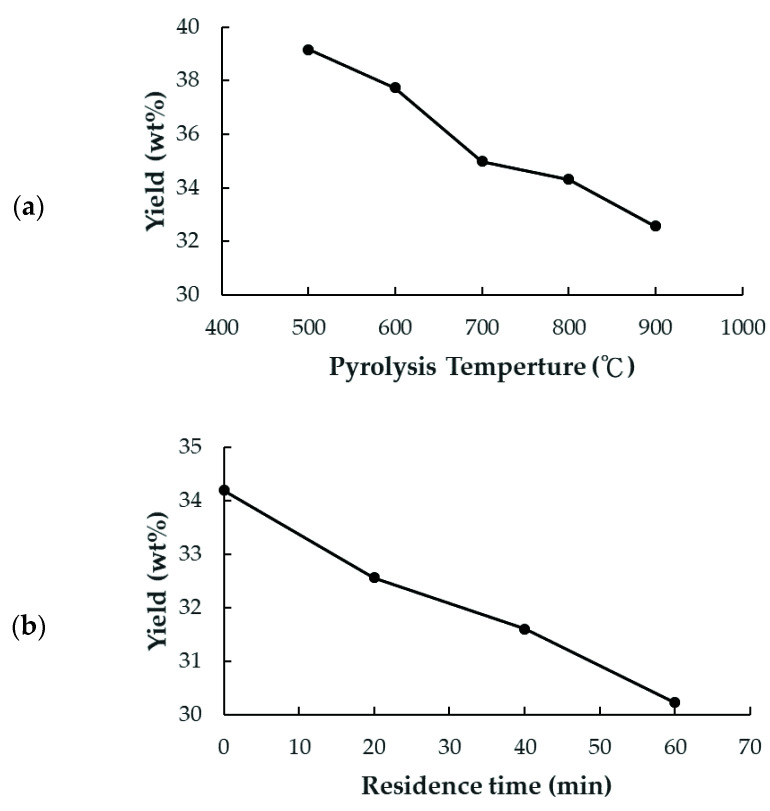
Variations in the yields of PP-BC products produced at (**a**) different pyrolysis temperatures for a residence time of 20 min, and (**b**) various residence times in 900 °C.

Figure 2 depicts the variations in the yields of the PP-BC products with a pyrolysis temperature for a residence time of 20 min and for a residence time in 900 °C. Obviously, the yields decreased with an increasing pyrolysis temperature from 39.15 wt% at 500 °C to 30.23 wt% at 900 °C (Figure 2a). This decrease in yield should be attributed to the serious charring reactions of lignocellulosic biomass at higher temperatures, leading to more loss of volatiles or forming lower-molecular-weight organic compounds and released gases (e.g., CH_4_, H_2_, CO_2_, and CO). This result was consistent with the TGA curve (Figure 1). Similarly, increasing the residence time up to 60 min at a pyrolysis temperature (900 °C) resulted in a gradual decrease in the biochar yield from 34.45 wt% to 30.23 wt%, as shown in Figure 2b.

In this work, the pore properties of biochar materials refer to a specific surface area, pore volume, and pore size. Table 2 summarizes the pore properties of the PP-BC products, which were prepared between 800 and 900 °C for residence times of 0–60 min. It was found that the pore properties of the PP-BC products produced at a pyrolysis temperature of below 800 °C were very small. Based on the calculation methods (i.e., single point, BET, Langmuir, and *t*-plot), the data on specific surface areas and pore volumes in Table 2 indicate a consistent trend. Moreover, they implied that the smaller the average pore width, the larger the pore properties, such as specific areas and pore volumes. As the pyrolysis temperature increased from 800 to 900 °C for a residence time of 20 min, the data on the BET surface area of the resulting biochar products significantly jumped from 11.98 to 119.43 m^2^/g. In the case of a 60 min residence time, the BET surface area also increased from 32.34 to 133.40 m^2^/g. Based on the data in Table 2, the optimal production conditions for producing PP-BC may be found at a pyrolysis temperature of 900 °C and residence time of 20 min. Although the increasing trend in the BET surface area of the resulting biochar was consistent with the study by Wang et al. [20], the largest pore properties (e.g., S_BET_ = 323.8 m^2^/g) occurred with the biochar prepared at a pyrolysis temperature of 750 °C with a long residence time (i.e., 120 min). In contrast, this work performed the optimal biochar production at 900 °C for a residence time of 20 min. When compared to the previous study [45], it showed an abrupt increase in the BET surface area (14.1 and 277.1 m^2^/g, respectively) of soapberry pericarp-based biochar produced at 700 to 800 °C for a residence time of 20 min. On the other hand, the residence time also affected the pore properties of the resulting biochar products, which were produced at 900 °C by extending the residence time from 0 to 60 min. It showed a significant increase in the BET surface area from 1.02 to 133.40 m^2^/g. This finding should be attributed to the severe carbonization at high temperatures for longer residence times, especially for the span of 0 to 20 min. Assuming the pores are of cylindrical and uniform geometry, the average pore diameter was further calculated from the ratio of the total pore volume to the BET surface area [34]. Obviously, the smaller the average pore diameter, the larger the pore properties. The average pore diameters of the SP-BC-900-20 and SP-BC-900-60 products were close to the boundary limit (2.0 nm) between micropores (pore diameter of <2.0 nm) and mesopores (pore diameter of 2.0–50.0 nm). Thus, both the micropores and mesopores should be presented in the porous biochar products, forming the fused ring structures of aromatic carbon matrices.

**Figure 3:**
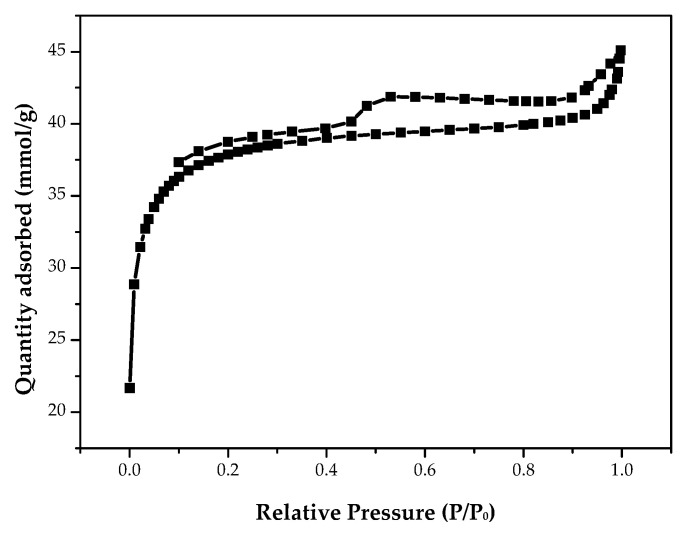
N_2_ adsorption–desorption isotherms of the biochar product (PP-BC-900-20).

**Figure 4:**
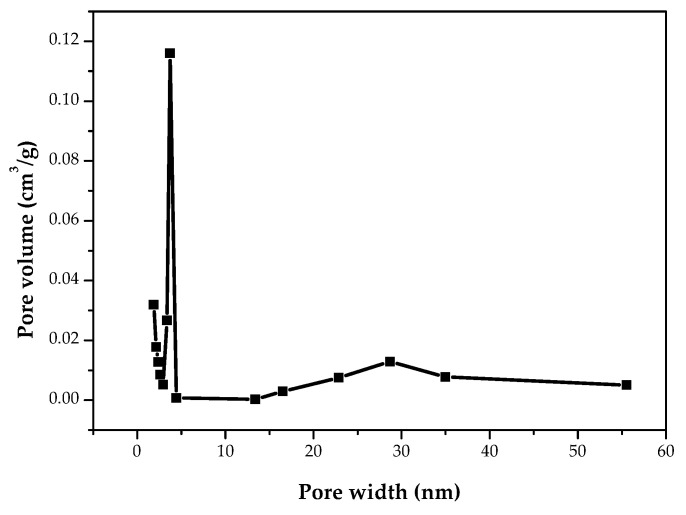
Pore size distribution curve of the biochar product (PP-BC-900-20).

**Figure 5:**
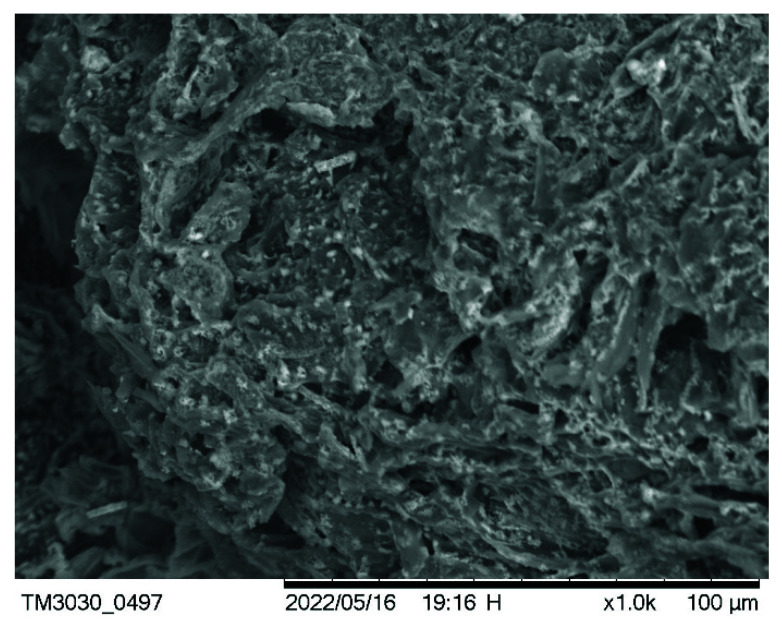
SEM image (×1000) of the biochar product for PP-BC-900-20.

It is well known that the pore structures of porous materials can be based on their adsorption–desorption isotherms [33,34]. Figure 3 shows the nitrogen adsorption–desorption isotherms of the PP-BC-900-20 product at −196 °C. From the isotherm shape, this biochar product obviously exhibited Type I characteristics because its high uptake by micropore filling was observed at very low relative pressures (P/P_0_ < 0.1) [34]. In addition, the resulting product also exhibited a hysteresis loop from the adsorption branch to the desorption branch, which is typical for Type IV isotherms [34]. In this regard, both the micropores and mesopores are present in the biochar product. More consistently, the mesopores in the pore width range of 3.0 to 4.5 nm were clearly observed in its pore size distribution curve using the Barret, Joyner, and Halenda (BJH) method (Figure 4). However, the peak observed at approximately 4 nm did not reflect the exact pore properties of the biochar material, but rather, the pore size distribution was determined primarily by the nature of the nitrogen desorption isotherm [46]. As mentioned above, the biochar PP-BC-900-20 was recognized as the optimal product in this work (Table 2). Figure 5 only shows the porous texture on the surface of the optimal biochar product (i.e., PP-BC-900-20) by the use of SEM.

**Figure 6:**
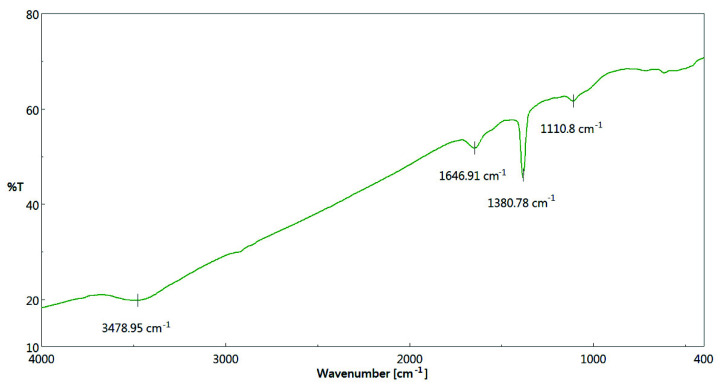
FTIR spectrum of the biochar product (PP-BC-900-20).

Since the oxygen complexes of biochar play a vital role in its polar nature (e.g., hydrophilicity) [47], the oxygen-containing functional groups on the surface of the biochar products were analyzed by Fourier transform infrared spectroscopy (FTIR). Due to the similar FTIR patterns of all biochar products, Figure 6 only illustrates the FTIR spectrum of the optimal biochar product (i.e., PP-BC-900-20) in the range of 400–4000 cm^−1^, which is discussed below [21,22]:

(1) The absorption peak at 3450 cm^−1^ in the region of 3300–3700 cm^−1^ should be assigned to the stretching vibration of the hydroxyl (O-H) group.
(2) The peak at approximately 1647 cm^−1^ could be associated with carbonyl (C=O) group stretching.
(3) The sharp peak at 1380 cm^−1^ could be due to C-O asymmetric stretching.
(4) The peak at 1110 cm^−1^ could be attributed to C-O-C aliphatic/ether stretching.
(5) Several weak peaks in the region of <1000 cm^−1^ were assigned to be due to C-H bending for aromatic out-of-plane deformations.

On the other hand, the elemental compositions of the biochar products and PP were detected by EDS, which showed that the main elements on the surface of the biochar products included carbon (>60 wt%) and oxygen (30–35 wt%), though differing amounts of carbon (~50 wt%) and oxygen (40–50 wt%) were present in the PP (Table 1). Thus, the PP-BC biochar product could be hydrophilic because of its richness in the oxygen-containing functional groups and ash minerals (e.g., carbonates, phosphates, sulfates, silica, and alumina) derived from PP (as seen in Table 1) [48].

### 3.3. Adsorption Performances of the PP-BC Product

**Figure 7:**
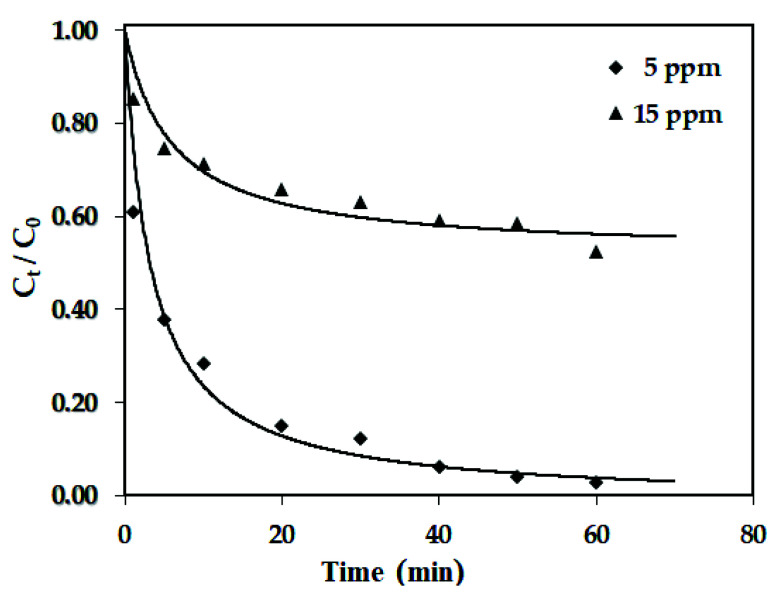
Variations of dimensionless MB concentrations (C*_t_*/C_0_) with adsorption times under various initial MB concentrations (i.e., PP-BC-900-20 dosage: 0.3 g/2 L, initial pH: 7.0, adsorption temperature: 25 °C); the symbols denote experimental data, and the full lines were obtained from the pseudo-second-order model using the fitted parameters (Table 3).

In order to test the adsorption performance of the biochar product, it was preliminarily used to serve as an adsorbent for the removal of cationic adsorbate (i.e., MB) from aqueous solutions due to its porous structure and hydrophilic features. Figure 7 depicts the variations of the dimensionless residual MB concentration (C*_t_*/C_0_) with adsorption times at specific adsorption conditions under the initial MB concentrations of 5 and 15 mg/L. As shown in Figure 7, the MB adsorption of the biochar has relatively fast kinetics, indicating a considerable interaction between the adsorbate and the adsorbent due to electrostatic attraction. This behavior was due to the driving force from the MB concentration gradient and the limited adsorption sites on the pores of the biochar. Table 3 summarizes the fitted values of the adsorption parameters, showing high correlation coefficients (>0.98) for this kinetic model. This result was also in accordance with the previous studies on the adsorption of MB onto the biochar products of from cocoa pod husk [49] and dairy cow manure [50]. As the initial MB concentration (i.e., C_0_) increased, more MB molecules were adsorbed onto the biochar at an equilibrium condition without depleting its adsorption, resulting in the fitted adsorption capacity that (*q_e_*) increased. However, the adsorption rate constant *k* and the initial adsorption rate *h* showed a decreasing trend at larger initial MB concentrations because of the limited available adsorption sites. When compared to the MB adsorption of the cow-manure-based biochar under the same adsorption conditions [50], it showed higher *q_e_* values due to its larger BET surface area (i.e., 294 m^2^/g).

## 4. Conclusions

This work compared the pore properties and adsorption performances of biochar products produced from pineapple peel (PP) at elevated pyrolysis temperatures. The conclusions are summarized below:

- The dried PP biomass has a relatively higher ash content (8.28 wt%) than that of common biomass husks, but it has a high combustible content comprising volatile matter (73.23 wt%) and fixed carbon (18.49 wt%).
- As the pyrolysis temperature increased from 800 to 900 °C for a residence time of 20 min, the data on the BET surface area of the resulting biochar products significantly jumped from 11.98 to 119.43 m^2^/g. At a residence time of 60 min, the BET surface area also increased from 32.34 to 133.40 m^2^/g.
- From the data on the nitrogen adsorption–desorption isotherms and pore size distribution, both micropores (pore diameters of <2.0 nm) and mesopores (pore diameters of 2.0–50.0 nm) are present in the PP-based biochar products.
- Due to its good fittings in the pseudo-second-order model and its hydrophilic nature as seen in the Fourier Transform infrared spectroscopy (FTIR), the resulting biochar could be a porous material to be used for the effective removal of cationic compounds (i.e., methylene blue (MB)) from liquid phases.

However, it would be helpful to study the activation of PP-based biochar (for enhancing its pore properties) and the effects of its other adsorption parameters (i.e., pH, the mass of adsorbent, and temperature) on the removal of MB from aqueous solutions by using PP-based carbon materials.

## Figures and Tables

**Table 1:** Thermochemical properties of pineapple peel (PP).

Properties ^a^	Value
Proximate analysis ^b^	
Ash (wt%)	8.28 ± 0.36
Volatile matter (wt%)	73.23 ± 0.71
Fixed carbon ^c^ (wt%)	18.49
Elemental composition ^d^	
Carbon (wt%)	50.49
Oxygen (wt%)	48.25
Sodium (wt%)	0.04
Magnesium (wt%)	0.19
Aluminum (wt%)	0.04
Silicon (wt%)	0.14
Phosphorus (wt%)	0.33
Sulfur (wt%)	0.47
Calcium (wt%)	0.05
Calorific value (MJ/kg) ^b^	18.02 ± 0.24

^a^ On a dry basis; ^b^ the mean ± standard deviation of the three determinations; ^c^ by difference; ^d^ using the EDS for the determinations of elemental compositions.

**Table 2:** Pore properties of resulting biochar products.

Pore Property	PP-BC-800-20	PP-BC-800-60	PP-BC-900-00	PP-BC-900-20	PP-BC-900-60
Surface area (m^2^/g)					
Single point surface area ^a^	12.06	32.66	˂2	117.49	131.14
BET surface area ^b^	11.98	32.34	˂2	119.43	133.40
Langmuir surface area	17.69	47.63	˂2	184.88	194.05
*t*-plot micropore area ^c^	8.90	25.82	-- ^g^	88.53	107.56
*t*-plot external surface area ^d^	3.08	6.52	-- ^g^	30.90	25.84
Pore volume (cm^3^/g)					
Total pore volume ^e^	0.0119	0.00223	-- ^g^	0.0687	0.0731
*t*-plot micropore area ^c^	0.0046	0.00134	-- ^g^	0.0448	0.0545
Pore size (nm)					
Average pore width ^f^	3.99	2.76	-- ^g^	2.30	2.19

^a^ Calculated at a relative pressure of 0.30 using the BET method; ^b^ calculated at a relative pressure range of 0.06–0.30 (15 points) using the BET method; ^c^ using the *t*-plot method; ^d^ obtained by subtracting the *t*-plot micropore area from the BET surface area; ^e^ calculated at a relative pressure of approximately 0.995 (i.e., saturated adsorption); ^f^ obtained by the ratio of the total pore volume (V_t_) to the BET surface area (S_BET_) (i.e., average pore width = 4 × V_t_/S_BET_), and assuming the cylindrical pore geometry; ^g^ these values can be not obtained or calculated from the analytical instruments due to the very limited pore properties.

**Table 3:** Pseudo-second-order model parameters for MB adsorption onto PP-BC-900-20 at various initial MB concentrations (^a^).

Initial MB Concentration (mg/L or ppm)	*k* (g/(mg·min))	*q_e_* (mg/g)	Correlation Coefficient	*t*_1/2_ (min)	*h* (mg/(g·min))
5	0.0151	20.28	0.998	3.21	6.21
15	0.0059	28.82	0.981	5.88	4.90

^a^ Process conditions: PP-BC-900-20 dosage = 0.3 g/2 L, initial pH = 7.0, adsorption temperature = 25 °C.

## Data Availability

Not applicable.
